
JOURNAL INFORMATION
==============================
NLM Title Abbreviation: ScientificWorldJournal
Journal ID: ScientificWorldJournal
NLM Journal ID: 101131163
Journal ID: TSWJ

The Scientific World Journal

ISSN: 2356-6140
EISSN: 1537-744X
Publisher: Wiley

ARTICLE INFORMATION
==============================
PMCID: PMC6084104
PMID: 30147470
DOI: 10.1100/tsw.2001.157
Article ID: 284921
Article version: 1
Subjects: Short Report

The Bloom Syndrome Protein BLM Is Selectively Cleaved during Apoptotic Cell Death

Bischof Oliver 1 *
Galande Sanjeev 2
Kohwi-Shigematsu Terumi 2
Campisi Judith 2
Dejean Anne 1
1 Dept. Retrovirus, Unité Recombinaison et Expression Génétique, Institut Pasteur, France
2 Life Sciences Division, Lawrence Berkeley National Laboratory, 1 Cyclotron Road, Berkeley CA 94720, USA
*Oliver Bischof: obischof@pasteur.fr
Publication date: 2001
Electronic publication date: 2001 Nov 9
Volume: 1
First page: 34
Last page: 34
Copyright: Copyright © 2001 Oliver Bischof et al.
Copyright year: 2001
License: This is an open access article distributed under the Creative Commons Attribution License, which permits unrestricted use, distribution, and reproduction in any medium, provided the original work is properly cited.
License URL: https://creativecommons.org/licenses/by/3.0/

Funding: National Institutes of Health GM59901 National Institutes of Health AG11658 U.S. Department of Energy DE-AC03-76SF00098

==============================
Miami *Nature Biotechnology* Short Reports
*TheScientificWorld* (2001) 1(S3), 34SR
ISSN 1532-2246; DOI 10.1100/tsw.2001.157

### THE BLOOM SYNDROME PROTEIN BLM IS SELECTIVELY CLEAVED DURING APOPTOTIC CELL DEATH

Oliver Bischof*, Sanjeev Galande‡, Terumi Kohwi-Shigematsu‡, Judith Campisi‡, Anne Dejean*

Dept. Retrovirus, Unité Recombinaison et Expression Génétique, Institut Pasteur, France; ‡ Life Sciences Division, Lawrence Berkeley National Laboratory, 1 Cyclotron Road, Berkeley CA 94720, USA
* obischof@pasteur.fr

**INTRODUCTION.** Bloom syndrome (BS) is an autosomal recessive disorder characterized by a high incidence of cancer and genomic instability. BLM, the protein defective in BS, is a RECQ-like helicase that is presumed to function in mammalian DNA replication, recombination or repair. Because of its importance in maintaining genomic stability, we asked whether BLM was among the proteins targeted for selective degradation during the execution phase of apoptosis as has been shown recently for PARP and ATM (1, 2), among others. We show here that BLM is rapidly cleaved in cells undergoing apoptosis.

**RESULTS.** BLM was cleaved to 47 and 110 kDa major fragments, with kinetics similar to the apoptotic cleavage of poly-ADP ribose polymerase (only ΔBLM of 47 kDa is shown in the Fig.). BLM cleavage was prevented by a caspase 3 inhibitor, and did not occur in caspase 3-deficient cells. Moreover, recombinant BLM was cleaved to 47 and 110 kDa fragments by caspase 3, but not caspase 6, *in vitro*. The caspase 3 recognition sequence 412TEVD415 was verified by mutating aspartate-415 to glycine, and showing that this mutation rendered BLM resistant to caspase 3 cleavage. Apoptotic cleavage did not abolish the BLM helicase activity, but abolished BLM nuclear foci and the association of BLM with condensed DNA and the insoluble matrix. The results suggest that BLM is an early selected target during the execution of apoptosis.

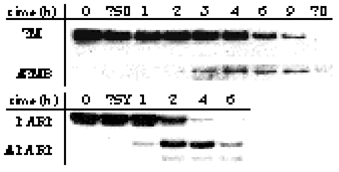

**DISCUSSION.** Caspases are important for both the initiation and execution phases of apoptosis (3-5). At present, there is a need to identify caspase substrates in order to understand how caspases execute apoptosis. In recent years, a number of caspase substrates have been identified (5). Here, we show that BLM is a substrate for the executioner caspase 3, *in vitro*

and *in vivo*. Immunostaining and biochemical fractionation showed that cleaved BLM lost its characteristic punctate nuclear localization, detached from an insoluble substructure, and dissociated from condensed DNA. Cleavage and loss of localization very likely obliterates the *in vivo* function of BLM. The potential function of BLM in DNA repair suggests that its cleavage and redistribution may aid nuclear disassembly and prevent the complex in which it resides from participating in the repair of fragmented DNA molecules generated by caspase-activated DNAse I.

**ACKNOWLEDGEMENT**. This work was supported by Marie-Curie Fellowship BMH4-CT98-5129 to OB, grants from the National Institutes of Health GM59901 to TKS and AG11658 to JC, and a program project grant from the U. S. Department of Energy, under contract DE-AC03-76SF00098 to the University of California.

**REFERENCES.**

1. Lazebnik, Y.A., Kaufmann, S.H., Desnoyers, S., Poirier, G.G., and Earnshaw, W.C. (1994) Nature 371, 346-347
2. Smith, G.C., di Fagagna, F., Lakin, N.D., and Jackson, S.P. (1999) Mol. Cell. Biol. 19, 6076-6084
3. Cohen, G.M. (1997) Biochem. J. 326, 1-16
4. Budihardjo, I., Oliver, H., Lutter, M., Luo, X., and Wang, X. (1999) Annu. Rev. Cell Dev. Biol. 15, 269-290
5. Earnshaw, W.C., Martins, L.M., and Kaufman, S.H. (1999) Annu. Rev. Biochem. 68, 383-424
